# Current progress of rehabilitative strategies in stem cell therapy for spinal cord injury: a review

**DOI:** 10.1038/s41536-021-00191-7

**Published:** 2021-11-25

**Authors:** Syoichi Tashiro, Osahiko Tsuji, Munehisa Shinozaki, Takahiro Shibata, Takashi Yoshida, Yohei Tomioka, Kei Unai, Takahiro Kondo, Go Itakura, Yoshiomi Kobayashi, Akimasa Yasuda, Satoshi Nori, Kanehiro Fujiyoshi, Narihito Nagoshi, Michiyuki Kawakami, Osamu Uemura, Shin Yamada, Tetsuya Tsuji, Hideyuki Okano, Masaya Nakamura

**Affiliations:** 1 Department of Rehabilitation Medicine, Keio University School of Medicine, Shinjuku, Tokyo Japan; 2 Department of Rehabilitation Medicine, Kyorin University School of Medicine, Mitaka, Tokyo Japan; 3 Department of Orthopaedic Surgery, Keio University School of Medicine, Shinjuku, Tokyo Japan; 4 Department of Physiology, Keio University School of Medicine, Shinjuku, Tokyo Japan; 5 Department of Rehabilitation, Murayama Medical Center, Musashi-Murayama, Tokyo Japan; 6 Department of Orthopaedic Surgery, Murayama Medical Center, Musashi-Murayama, Tokyo Japan; 7 Department of Orthopaedic surgery, National Defense Medical College, Tokorozawa, Saitama Japan

**Keywords:** Trauma, Spinal cord injury

## Abstract

Stem cell-based regenerative therapy has opened an avenue for functional recovery of patients with spinal cord injury (SCI). Regenerative rehabilitation is attracting wide attention owing to its synergistic effects, feasibility, non-invasiveness, and diverse and systemic properties. In this review article, we summarize the features of rehabilitation, describe the mechanism of combinatorial treatment, and discuss regenerative rehabilitation in the context of SCI. Although conventional rehabilitative methods have commonly been implemented alone, especially in studies of acute-to-subacute SCI, the combinatorial effects of intensive and advanced methods, including various neurorehabilitative approaches, have also been reported. Separating the concept of combined rehabilitation from regenerative rehabilitation, we suggest that the main roles of regenerative rehabilitation can be categorized as conditioning/reconditioning, functional training, and physical exercise, all of which are indispensable for enhancing functional recovery achieved using stem cell therapies.

## Introduction

Spinal cord injury (SCI) results in various neurological sequelae in the motor, sensory, and autonomic systems. There is no treatment in the strict sense, only approaches to reduce secondary damage acute SCI, including surgical procedures to restabilize and decompress the spinal cord and to augment blood pressure^1^. Rehabilitative therapies are performed after these procedures, but the injured spinal cord exhibits only a small degree of plasticity and functional recovery^2^. For more than two decades, stem cell-based regenerative therapy has been investigated as a state-of-the-art treatment that is expected to change the prognosis after SCI. Both cellular graft sources, including olfactory ensheathing cells (OECs), mesenchymal stem cells (MSCs), and neural stem/progenitor cells (NS/PCs), and tissue graft sources, including peripheral nerve and olfactory mucosa, have been investigated. Although some of these methods have proceeded to the clinical trial stage, the transplanted stem cells do not always work as we expected and thus the current data only show limited functional recovery. One factor that markedly affects the therapeutic potential is the microenvironment, which changes over time after injury^3^. Although many researchers reported that stem cell therapies have significant effects in the early phases, they lose their therapeutic potential in the chronic phase as neuronal plasticity decreases^1,4–6^. There are 50-fold more patients in the chronic phase than in the acute-to-subacute phase; therefore, it is crucial to establish strategies that can be used to treat the chronically injured spinal cord^7^.

Recently, a strategy to combine optimal rehabilitation with regenerative treatments, called regenerative rehabilitation^8,9^, has been proposed based upon preclinical^10–12^ and clinical^13–15^ research that reported a variety of mechanisms and effects on physical functions including muscle strength, gait, and the ability to perform activities of daily living (ADL)^9,16,17^. This concept is concisely defined as “*The application of rehabilitation protocols and principles together with regenerative medicine therapeutics toward the goal of optimizing functional recovery through tissue regeneration, remodeling, or repair*”^8^. Rehabilitation is suggested to promote functional integration of the graft and host neuronal system when combined with stem cell therapies^2^. However, to the best of our knowledge, regenerative rehabilitation following SCI has not been structurally summarized because research in this area is in its infancy. Therefore, this review provides an overview of the mechanisms of rehabilitation performed in combination with stem cell therapies and describes the concepts of clinical regenerative rehabilitation. In addition, we briefly introduce rehabilitative strategies more generally, independent of their combinatorial use with regenerative therapies, to provide a more integrated discussion of this area. Although regenerative medicine encompasses various treatments including cell, tissue, and organ replacement, cytokine therapy, and neurorehabilitative approaches to induce regeneration of impaired body parts and/or systems^18,19^, this review specifically deals with stem cell transplantation therapies. Rehabilitation includes physical treatments utilizing electrical stimulation, magnetic stimulation, and infrared and ultrasound treatments. Here, we focus on exercise training and physical therapies used in combination with exercise.

We searched studies in the Web of Science (BIOSIS), Medline (via PubMed), Scopus, and ProQuest databases from the beginning of 1981 to 1 July 2021. Keywords related to “spinal cord injury” and “transplantation” in combination with terms related to “rehabilitation” and “training” were used in searches for preclinical research. In addition, although some functional assessments of forelimbs require training, this training is often not described in the abstract. Therefore, we closely checked the methods sections of the studies chosen using keywords related to “forelimb” instead of “rehabilitation” to determine whether rehabilitative training was implemented. Studies in which the post-therapeutic training frequency was no less than three times per week were assembled into a table. Keywords related to “spinal cord injury” and “transplantation” limited to clinical research were applied to searches for clinical studies.

## SCI rehabilitation models in preclinical studies

Owing to technical limitations, no consensus has been reached regarding rehabilitative methods in experimental animals. Interventional studies targeting hindlimb and gait function are predominantly conducted in rodents, while few studies have used minipigs, felines, and non-human primates. Many of these studies applied the thoracic cord injury model and implemented physical training including hindlimb motion due to the advantages of more accessible assessment and a higher survival rate, together with the applicability of various SCI types and their severity. Quadrupedal treadmill training was adopted in most mouse studies, while both bipedal and quadrupedal treadmills were used in rat models. Body-weight supporting apparatus is frequently applied to enable the training in animals with severe impairment. Cycling, swimming, and climbing training routines were also sometimes used in rodents. The most suitable rehabilitative method might differ between models. Quadrupedal training is called physiological gait training because it enables coordination between the forelimbs and hindlimbs. A validated and standardized training protocol was recently reported in moderate contusive SCI model mice^20^. By stimulating reorganization of rostrocaudal spinal inter-neuronal networks, a research group reported better functional recovery with quadrupedal training than with bipedal gait training in a hemisection rat model^21^. On the other hand, bipedal training has advantages because it explicitly trains the paretic limbs even in severe SCI animal models that tend to use forelimbs more than hindlimbs in quadrupedal gaits^18^, and it may be better suited for investigating the effects on the local spinal network. Cycling training involves rhythmic sensory-motor training and promotes right–left coordination along with range-of-motion exercises^22^. Some researchers used swimming training for incomplete SCI model rats because it is a natural behavior of this species. Although it requires simple apparatus, controlling an appropriate exercise load is difficult. Importantly, specific training paradigms to encourage voluntary stepping are more effective than entirely passive stepping^18,23^. Instrumental training promotes activity-based learning and suppresses maladaptive plasticity^24^. By contrast, while it remains controversial, some reports suggest there are unfavorable training conditions that may lead to the development of hypersensitivity, such as delayed initiation of running wheel training resulting in aberrant nociceptive plasticity for cervical hemi-contusion model rats^25^ and early initiation of passive mechanical training for moderately thoracic cord contusion model rats^26^. In addition, researchers have reported a trade-off relationship, the deterioration of a specific function secondary to other training targeting functions, between locomotor and forelimb dexterity tasks in rats with cervical dorsolateral quadrant lesion^27,28^ and between standing and stepping abilities in rats with dorsal rhizotomy^29^. This emphasizes the importance of designing optimal therapeutic strategies corresponding to the impairment model^22^.

Forelimb functional recovery in animals with cervical SCI has also been investigated. Although gait analysis, climbing tasks, the cylinder test, grip strength, and object manipulation, including food-pellet reaching tasks, are applied as assessment measures, reaching tasks require training to evaluate the acquisition of dexterity^30–35^. A single-pellet reaching task, Whishaw reaching, involves horizontal reaching training, and a clear relationship between training intensity and improvement was recently shown in rats with cervical dorsolateral quadrant lesion^36^. The modified Montoya staircase task involves training for perpendicular reaching of each hand independently^37^. Compared with gait training, such task-specific training has advantages including a lack of confounding by off-task home cage self-training that overrides the training effect^38^. In addition, forelimb function has been actively investigated not only with SCI models, but also in the context of the specific contribution of a tract(s) and propriospinal neurons^39^.

Although the number of rehabilitative training animal models is still limited^40^, a growing number of studies have demonstrated functional recovery induced by rehabilitative training. With regard to motor function, a variety of changes have been reported, including regeneration and reorganization of intra-spinal^27,41–43^ and spinal descending circuits^44^, enforcement of synaptic function^45^, axonal regeneration, and exercise-dependent plasticity^46^, improvements of motor control through the restoration of spinal inhibitory capacity^18,47^, sensory-motor integration, and supra-spinal control^48^. Expression of various neurotrophic factors^49,50^, growth factors^12,51^, and both excitatory and inhibitory molecules^18,47^ has been suggested to be involved in these changes. Neurotrophic factors have been proposed to promote neural plasticity, vascularization, and neuroprotection^8^. Furthermore, treadmill training reportedly promotes proliferation and migration of ependymal cells, which are considered to be a source of neural stem cells, in thoracic cord clip-compression model rats^52^. Although it is reported that ependymal cells do not proliferate after injury in the adult human spinal cord^53^, another group reported that they are activated in some humans of a certain age^54^ and in animal models including mouse transection SCI^55,56^. A similar beneficial effect might be induced by rehabilitation in combination with stem cell transplantation.

Another critical role of rehabilitation is to prevent and ameliorate the negative impact of disuse due to impairments^57^. Muscle volume and function decrease after SCI, and the dominant muscular fiber type changes^58^. Importantly, disuse muscle atrophy is also reported to promote motor neuronal degeneration^59^. Moreover, although it is often overlooked in preclinical studies of chronic SCI, disuse-induced functional deterioration is speculated to suppress or even mask the beneficial effect of specific treatments^11^.

## Methodology of regenerative rehabilitation in preclinical studies

Although rehabilitation is commonly performed after cell therapy in human patients, preclinical studies are particularly important for the following purposes: (1) to assess the synergistic effect of transplantation and rehabilitation and (2) to broaden the applications in patients with chronic SCI in whom stem cell therapy is ineffective when applied alone^2^. Very few preclinical studies have investigated the combinatorial effects of stem cell therapy and rehabilitation regardless of chronicity (Table 1, Supplementary Table 1). Most studies targeted acute and subacute SCI, and only one or two research groups have investigated combinatorial treatment strategies, except for observational studies incorporating very few animals^10,11,22^. Rehabilitation training was usually initiated 2–7 days after transplantation, while the training period varied from 4 weeks to several months. A frequency of 5–7 days per week is adopted in most regimens, although a 3-day regimen was tested for rats with mild thoracic cord contusion SCI in an earlier study^60^. The time duration varied from 20 to 60 min per day in most studies, but training for a shorter duration was applied in a few cases^61^. Some researchers incorporated a pretraining period of ~1 week before the transplantation procedure and the main training period followed this. The purpose of pretraining is to recondition the animals’ body function after a long period of low activity following injury and to habituate the animals for training^10–12,62^. However, to our knowledge, none of these methods is standardized in terms of appropriate load, duration, and methodology.

**Table 1:** SCI rehabilitation in preclinical studies on stem cell therapies.

Study	Rehabilitation
2021 Sun W.M.^70^, Rhesus monkeys, *N* = 4. Acute Tp (0 DPI), T8 hemisection	Regular stretching, standing with supportive chair, positioning, chair supported treadmill ambulation. Two 20-min sessions/day, 5 d/wk. Untrained condition was not investigated.
2021 Prager J.^71^, WH rats, *N* = 23. Acute Tp (0 DPI), C3 dorsal column crush	Forepaw reaching; started at 7 DPI, 1 h/day, 5 d/wk, for 8 wks. Untrained condition was not investigated.
2020 Younsi A.^67^, WH rats, *N* = 70. Subacute Tp (10 DPI), C6 moderate contusion	Quadrupedal treadmill; started at DPI, 20 min/day, for 6 wks.
2020 Dugan E.A.^74^, SD rats, *N* = 48. Late subacute Tp (28 DPI), T6-7 clip contusion	Quadrupedal 8 degree incline treadmill in the ramping protocol; started at 5 or 35 DPI, 20 min/day, 5 d/wk, for 4 wks.
2020 Massoto T.B.^66^, C57 B6j mice, *N* = 40. Subacute Tp (7 DPI), T9 clip contusion	Quadrupedal treadmill; started at 14 DPI, 10 min/day, 3 d/wk, for 8 wks.
2018 Thornton MA.^104^, SD rats, *N* = 10. Acute Tp (0 DPI), T6-7 transection	Climb training with 40 Hz ES at a 95% of motor threshold; started at 28 DPI, 20 min/day, 3/week, for 6 mo. Conditions without training or ES were not evaluated.
2018 Tashiro S.^10^, C57 B6j mice, *N* = 45. Chronic Tp (49 DPI), T9 severe contusion	Bipedal treadmill; started at 52 DPI, 20 min/day, 5 d/wk, for 8 wks. Pretraining for 1 wk started at 42 DPI
2017 Theisen C.C.^22^, SD rats, *N* = 45. Chronic Tp (42 DPI), Late subacute rehab (35 DPI) T12 transection	Cycling exercise: 30 min/day; the Delayed group started at 35 DPI for 6 wks, Acute group started at 5 DPI for 10 wks.
2016 Nicola F.C.^61^, WH rats, *N* = 54. Acute Tp (0 DPI), T9 moderate contusion	Quadrupedal treadmill; started at 3 DPI, 10 min/day, 5 d/wk, for 6 wks. Pretraining for 1 wk
2016 Tashiro S.^11^, C57 B6j mice, *N* = 80. Chronic Tp (49 DPI), T9 severe contusion	Bipedal treadmill; started at 52 DPI, 20 min/day, 5 d/wk, for 8 wks. Pretraining for 1 wk from 42 DPI
2016 Sachdeva R.^63^, SD rats, *N* = 45. Acute Tp (0 DPI), T12 transection	Cycling exercise at 45 rpm; started at 5 DPI, 30 min/day, 5 d/wk, for 4 wks.
2015 Dugan E.A.^105^, SD rats, *N* = 64. Acute Tp (0 DPI), T10 transection	Passive quadrupedal cycling; started at 5 DPI, 60 min/day (two 30-min sessions with a 10-min rest), 5 days/wk, for 12 wks.
2014 Hwang D.H.^12^, SD rats, *N* = 184. Subacute Tp (7 DPI), T9 moderate contusion	Quadrupedal treadmill; started at 10 DPI, 60 min/day, 6 d/wk, for 8 wks. Pretraining for 1 wk
2013 Sun T.^65^, SD rats, *N* = 40. Subacute Tp (14 DPI), T10 moderate contusion	Bipedal treadmill; started at 21 DPI, 20 ± 10 min/day, for 10 wks.
2011 Takeoka A.^64^, WH rats, *N* = 41. Acute Tp (0 DPI), T9 transection	Bipedal step and treadmill gait; started at 14 DPI, 20 min/day, for 7.5 mo.
2008 Kubasak M.D.^62^, WH rats, *N* = 38. Acute Tp (0 DPI), T9 transection	Bipedal treadmill stepping; 5 min in the first week, and increased by 5 min each week up to 20 min/day, for 6 mo in total. Untrained condition was not investigated histologically.
2008 Carvalho K.A.T.^106^, WH rats, *N* = 48. Acute Tp (2 DPI), T9/10 mild contusion	Swimming with support; 60 min/day, 6 d/wk, for 6 wks.
2006 Yoshihara H.^60^, SD rats, *N* = 26. Subacute Tp (9 DPI), T9-10 mild contusion	Passive hindlimb bicycling at 45 rpm; started at 11–12 DPI, 60 min/day (two 30-min sessions with a 10-min rest), 3 d/wk, for 3 mo.
2006 Lynskey J.V.^107^, SD rats, *N* = 84. Subacute Tp (14 DPI), C5/6 over-hemisection	Direct skilled target reaching; started at 42 DPI, 5–10 min, for 5 days. Untrained condition was not investigated.
2005 Keyvan-Fouladi N.^72^, Albino Swiss rats, *N* = 46. Chronic Tp (56 DPI), C1/2 corticospinal tract lesion	Direct forepaw reaching; started at 59 DPI, 50 retrieval/limb, 3/wk, for 8 wks. Untrained condition was not investigated.
2005 Ruitenberg M.J.^108^, Fischer rats, *N* = 29. Chronic Tp (56 DPI), C4 dorsal hemisection	Direct forepaw reaching; started at 70 DPI, 3/wk, for 10 wks. Untrained condition was not investigated.
2003 Keyvan-Fouladi N.^109^, Albino Swiss rats, *N* = 54. Chronic Tp (56 DPI), cervical-dorsal hemisection.	Direct forepaw reaching; started at 59 DPI, 50 retrieval/limb, 3/wk, for 8 wks. Untrained condition was not investigated.

*DPI* days post injury, *ES* epidural stimulation, *SD rats* Sprague Dawley rats, *Tp* transplantation, *WH rats* Wistar Hannover rat.

In the early study of the combinatorial treatment in 2006, Yoshihara et al.^60^ did not observe any remarkable effect on rats with mild contusive thoracic SCI who received a subacute intramedullary injection of bone marrow-derived MSCs when hindlimb cycling exercise was applied at a frequency of three times per week. In 2016, Sachdeva et al.^63^ applied a similar training method with a higher frequency (five times per week) in spinal cord-transected rats who received a peripheral nerve graft. They found that cycling exercise strongly facilitated regeneration by propriospinal, but not sensory, neurons, accompanied by increases in mRNA expression of regeneration-associated genes. Regarding other studies that applied minimal doses, Nicola et al.^61^ did not find any additional effect of 10 min/day training on rats with mild thoracic SCI. Kubasak et al.^62^ applied incremental doses from 5 to 20 min/day and showed that functional recovery was significantly enhanced in a transection model. These results imply that rehabilitative treatments used in combination with stem cell therapy have a minimum dose threshold and that an appropriate method or strategy is required to elicit beneficial effects. Further investigations are needed to determine the appropriate dose, intensity, and training method for each SCI model.

## Mechanisms of regenerative rehabilitation in preclinical studies

**Fig. 1:**
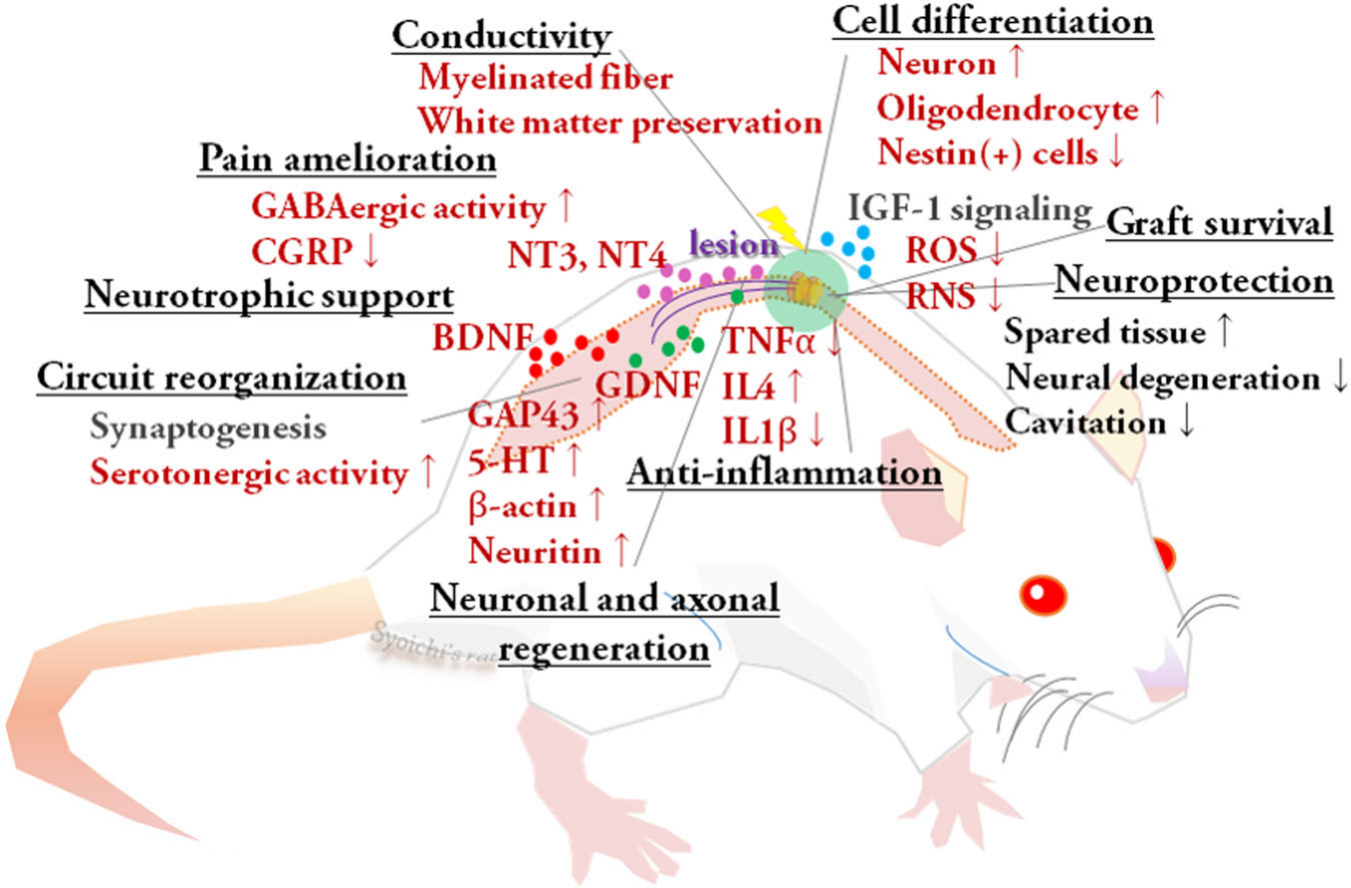
Mechanisms of regenerative rehabilitation revealed in preclinical studies. The mechanisms of regenerative rehabilitation are briefly summarized in this scheme. Studies lacking comparisons of trained and untrained groups and studies including only a few animals were excluded. *BDNF* brain-derived neurotrophic factor, *CGRP* calcitonin gene-related peptide, *GAP43* growth-associated protein 43, *GDNF* glial cell line-derived neurotrophic factor, *IGF-1* insulin-like growth factor, *IL1β/4* interleukin 1β/4, *GABA* gamma aminobutyric acid, *NT-3/-4* neurotrophic factor 3/4, *RNS* reactive nitrogen species, *ROS* reactive oxygen species, *TNFα* tumor necrosis factor α, *5-HT* 5-hydroxytryptamine.

With regard to the behavioral, histological, and functional aspects of hosts with acute and subacute SCI, Takeoka et al. reported that partial body-weight-supported treadmill training (BWSTT) gait exercise enhanced the effect of OEC grafts in super acute thoracic cord-transected rats. They observed that combinatorial treatments induced a fourfold increase in regenerating axons within the caudal stump of the transected spinal cord, and consequently improved hindlimb function and electrophysiological states. In addition, they found that the combinatorial effect was preserved after re-transection of the spinal cord, indicating that regenerative rehabilitation promoted the reorganization of lumbosacral circuits^64^. Sun et al. subacutely transplanted both OECs and Schwann cells into a rat with moderate contusion injury and performed bipedal BWSTT. While the activity of astrocyte-like OECs at the lesion site was not modified, they found that treadmill training significantly contributed to increased serotoninergic activity within lumbar enlargement. Locomotor function was significantly greater in the trained groups, and the effect was greater in the group that received combinatorial therapy than in the training-only group^65^. Hwang et al. acutely transplanted NS/PCs into the moderately contused rat spinal cord and then performed bipedal BWSTT for 8 weeks. The combinatorial approach significantly promoted graft survival and differentiation more into neurons and oligodendrocytes, which correlated with greater functional recovery than in the control groups. It also enhanced tissue protection, myelination, and restoration of serotonergic fiber innervation in the lumbar spinal cord, which the authors attributed to reduced stress caused by active oxygen or active nitrogen through insulin-like growth factor (IGF)-1 signaling^12^. In addition, they showed that combined rehabilitation contributed to upregulation of brain-derived neurotrophic factor (BDNF), glial cell line-derived neurotrophic factor (GDNF), and neurotrophic factor 3 (NT-3). Massoto et al. performed MSC transplantation in the acute phase and treadmill training for 8 weeks in mice with moderate clip-compression injury. They reported better functional recovery together with a larger area of white matter, more myelinated fibers, and fewer microcavitations and degenerating nerve fibers, along with significantly increased NT-4 expression^66^. Younsi et al. subacutely transplanted NS/PCs into cervical contusive lesions of rats and then performed quadrupedal treadmill training for 6 weeks. Combined rehabilitation enhanced graft survival and promoted differentiation into neurons and oligodendrocytes. They further demonstrated better functional recovery together with improved myelination, descending tract regeneration, and tissue sparing following combinatorial treatment^67^. Sachdeva et al. performed cycling training in combination with super acute peripheral nerve grafting in a rat transection model. They found that exercise enhanced propriospinal neuronal regeneration and mRNA expression of growth-associated protein 43 (GAP43), β-actin, and Neuritin, which are responsible for neuronal regeneration, while no remarkable effect was observed on sensory neurons^63^. The mechanisms are briefly illustrated in Fig. 1.

Only some of the studies that performed the combinatorial treatment with transplantation and rehabilitation focused on training-specific effects. Consequently, these studies sometimes did not compare trained and untrained or less-trained animals. This was particularly true of studies of forelimb function that utilized pellet reaching tasks; training was usually implemented just as part of the assessment only before SCI^68^ or for a short duration before every assessment^69^. Whereas other studies implemented the task as regular rehabilitative training after transplantation, there was no untrained groups^70–72^. Thus, to our knowledge, the specific contribution of regenerative rehabilitation to forelimb functional recovery has not been reported, and the findings described above were all obtained with gait training. Overall, the mechanism of regenerative rehabilitation has not been completely elucidated.

## Regenerative rehabilitation for the refractory state of delayed (late subacute to chronic) phase SCI

A consensus study concluded that 6 weeks is the minimum timeframe required to reach the chronic phase of SCI in the rodent model^73^. While combinatorial treatment with rehabilitation is a favorable treatment option, only two studies investigated combinatorial treatment with cell transplantation and rehabilitation in the chronic phase of SCI if the 6-week rule is strictly applied^10,11^. Tashiro et al.^10,11^ studied motor and sensory recovery following transplantation in combination with rehabilitation in the chronic phase. They performed NS/PC transplantation followed by bipedal BWSTT for 8 weeks in severe contusive thoracic SCI mouse models. The mice displayed significant locomotor recovery, which corroborated the synergistic effect of the combinatorial treatment on axonal regeneration and synaptogenesis, as well as an increase in neuronal differentiation of the transplanted cells. The additive effects on immunohistological changes imply that serotoninergic activity was enhanced by transplantation and GABAergic activity was restored by rehabilitation^11^. They further reported amelioration of thermal allodynia and coarse touch-pressure hyperalgesia, and a reduction of calcitonin gene-related peptide-positive fibers, which function in the transmission of pain, together with upregulation of GABAergic activity in the posterior horn^10^. Although they demonstrated significant functional recovery upon combinatorial therapy, no pertinent differences were observed between the combinatorial treatment and rehabilitation alone groups. Thus, they concluded that an additional treatment(s) is needed to use stem cell therapies in patients with chronic SCI^11^. Theisen et al.^22^ investigated the effects of peripheral nerve grafting at 42 days post injury (DPI) in combination with cycling exercise in T12-transected rats and reported that spinal axon regeneration was enhanced after exercise. No significant difference was detected between the two exercise periods, namely, 6 weeks starting at 35 DPI and 10 weeks starting at 7 DPI. Although these outcomes were due to a combination of chronic transplantation and acute or late subacute rehabilitative interventions, we would classify the latter as chronic regenerative rehabilitation. The difference between late subacute and chronic phase SCI has not been determined in terms of rehabilitation studies. A study by the same laboratory further compared the results of chronic peripheral nerve grafting with acute interventions^63^ and concluded that exercise has no long-lasting or cumulative effect in terms of axonal regeneration.

Regarding late subacute interventions, Dugan et al. investigated the role of the local spinal inhibitory circuit upon GABAergic neuronal progenitor cell transplantation in thoracic cord clip-compression rat models. Cells were transplanted into the lumbar enlargement excluding the injury site at 28 DPI, and treadmill quadrupedal training with an incline of 8° was performed for 4 weeks. They demonstrated that neuropathic pain assessed by allodynia, hyperalgesia, and ongoing pain was ameliorated upon reduction of inflammation, as represented by upregulation of IL4 and downregulation of tumor necrosis factor α and interleukin 1β, together with increased BDNF expression. Moreover, combined rehabilitation in both the subacute and chronic phases significantly promoted the restoration of GABAergic activity, while transplantation alone did not induce remarkable changes^74^. These studies imply that the effects of combinatorial treatment with rehabilitation and stem cell transplantation in the chronic phase are not inferior to those in the subacute phase with regard to sensory recovery, but the effects of transplantation on motor function decrease over time.

## Progress of neurorehabilitation in clinics

Joint range-of-motion exercise, paretic and non-paretic muscle strengthening, systemic physical capacity training, and practice of basic motions, including gait and transfer, are clinically applied as conventional rehabilitation^75^. Great efforts have been made to develop more effective rehabilitative treatments for patients with SCI. Researchers have reported that intensive overground walking together with training of balance function^76^, BWSTT^77^, and training of hand and reaching function^78^ effectively improve body function of SCI patients. It is noteworthy that multimodal rehabilitation can induce functional recovery even in patients with chronic motor-complete SCI^79^. It is suggested that the central nervous system (CNS) reorganizes itself during the acquisition, retention, and consolidation of motor skills. This concept is summarized as neurorehabilitation and neuroplasticity^80^. There might be such significant overlap between the chief mechanisms underlying the effects of stem cell therapies and neurorehabilitation that these two treatments show synergism^8,16^. Although most neurorehabilitative treatments are non-invasive, some require a neurosurgical procedure to implant epidural electrodes or an intrathecal drug administration system^81^.

Studies have reported the effects of various “peripheral” neurorehabilitative methods that target the peripheral sensorimotor system. Functional electrical stimulation (FES), including neuromuscular electrical stimulation (NMES), is a method to assist the impaired muscular function of patients using electrical stimulation. NMES assists the voluntary movements of patients by enhancing the intent of movements via timely and tuned electrical stimulation. It is utilized for the training of both gait and upper limb activities^82^. BWSTT and robot-assisted gait training (RAGT) improve the gait of patients in a more accessible, faster, and safer manner than regular overground training^83^. Some types of RAGT include additional functions such as FES^84^. Brain–computer interface is a new technology that connects specific brain signals of thoughts, perceptions, and motor intent to the output device, which provides a specific response that rewards functional training or a compensatory response to the impaired body function^85^.

On the other hand, the “central” neurorehabilitative method targeting the CNS has also been investigated with various means and strategies. Preclinical studies showed that direct current stimulation induces migration and proliferation of neural progenitor cells together with upregulation of neurotrophic factors^86^. In addition, both non-invasive transcutaneous and minimally invasive spinal/brain stimulation has been developed owing to their direct and harmless neuromodulatory properties^87,88^. Moreover, a series of studies revealed that combinatorial treatment with intrathecal medication and monoamine agonists enhances and manipulates the effect of electrical stimulation^89^. The brain-spine interface was recently reported in a primate model in which an electroencephalograph was applied to control epidural stimulation patterns^90^. Utilizing these multimodal neurorehabilitative approaches, Asboth et al. reported a novel rehabilitative treatment in which body-weight-supported RAGT was performed in combination with spinal epidural stimulation and treatment with serotoninergic and dopaminergic agonists in a preclinical study. They further observed reorganization of the efferent circuit cortico-reticulospinal circuit, which caused rerouting of cortical projections and contributed to functional recovery^44^.

## Regenerative rehabilitation in clinical studies: difference between acute-to-subacute phase and chronic phase

Several clinical studies of stem cell-based regenerative treatments with cellular or tissue grafting for human patients with SCI have been conducted worldwide. This section summarizes the features of rehabilitation in five studies of acute SCI, four studies of subacute SCI, 33 studies of chronic SCI, and nine studies in which the chronicity was not specified (Tables 2 and 3, Supplementary Tables 2 and 3). A group reported that intensive neurorehabilitation was applied for children who underwent transplantation involving surgical procedure^91^. It is noteworthy that only standard/conventional rehabilitation training was performed in studies of patients with acute and subacute SCI. This might be because preclinical research showed that stem cell therapies only induce significant functional improvement in the early phases. Therefore, researchers have no specific obligation to deliver stem cell therapy in combination with an intensive strategy or a neurorehabilitative approach using an advanced device, i.e., regenerative rehabilitation in a narrow sense.

**Table 2:** SCI rehabilitation in clinical studies on stem cell therapies involving acute/subacute patients.

Study information	Rehabilitation
2020 Chen W.^100^, Phase I, *N* = 7. Acute SCI, implantation, autologous bone marrow mononuclear cells loaded into NeuroRegen scaffolds	6 months of standard rehabilitation including routine care, hyperbaric oxygen therapy, neurotrophic therapy, acupuncture, neuromuscular electrical stimulation therapy, upper limb muscle strength training, self-care training, etc.
2020 Sharma A.^110^, Open-label, *N* = 180. Subacute to chronic SCI, intrathecal injection, autologous bone marrow mononuclear cells	Neurorehabilitation, including physiotherapy, occupational therapy, psychological interventions, and aquatic therapy. A home program under the supervision of professionals was recommended.
2017 Anderson K.D.^111^, Phase I, *N* = 6. Subacute SCI, intramedullary injection, autologous Schwann cells *A study to develop a home program was derived from this trial^101^	An inpatient standard medical rehabilitation, for 3–5 weeks pre-Tp, and for 6.6 ± 2.1 weeks post-Tp; 3 h/day, 5 d/wk.
2013 Liu J.^112^ *N* = 22., Subacute to chronic SCI, intrathecal injection, umbilical cord mesenchymal stem cells	A systemic individualized physical therapy
2007 Yoon S.H. ^113^, Phase: I/II, *N* = 35. Acute-to-chronic SCI: acute (<14 DPI): *N* = 17, Subacute (≥14 DPI, < 8 weeks): *N* = 6, Subacute-Chronic (≥8 weeks): *N* = 12 Intramedullary injection, autologous bone marrow cell	An active rehabilitation
Ravinovich S.S.^114^, Knoller N.^115^, Shin J.C.^116^, Karamouzian S.^117^, Chhabra H.S.^118^, Satti H.S.^119^, Hur J.W.^120^, Bansal H.^121^, Xiao Z.^122^	A standard rehabilitation
Pal R.^123^, Attar A.^124^, Kumar A.A.^125^, Saito F.^126,127^, Jones L.A.T.^128^, Lammertse D.P.^129^	No description about rehabilitation

*DPI* days post injury, *SCI* spinal cord injury, *Tp* transplantation.

**Table 3:** SCI rehabilitation in clinical studies on stem cell therapies for chronic patients.

Study information	Rehabilitation
2021 Gant K.L.^130^, *N* = 8 Injection (cavity-filling), autologous human SCs	Upper extremity circuit resistance training; 3 /wk Conditioning of lower extremities: FES and cycle ergometer; 2 /wk BWSTT with robot for AIS A or B, or overground locomotor skill for AIS C; biweekly.
2016 Zhu H.^97^, Yao L.^131^, *N* = 28 Intramedullary injection, umbilical cord blood-derived mononuclear cells	Intensive locomotor training; started at 14 POD, 6 h/day, 6 d/wk, for 3–6 mo
2016 Oh S.K.^93^, Phase III, *N* = 16 Subdural injection, autologous MSCs	(Pre) included only when no improvements with 3 months rehabilitation (Post) a standardized PT, twice a day, 6 d/wk, from 7 POD for 4 wks: tilt table (30 min), mat exercises (assistive ROM, overground functional activities, 30 min) FES.
2016 Iwatsuki K.^132^, *N* = 8 Olfactory mucosa grating with scar removal	A standard PT to encourage function below the lesion, enabling walking training as soon as possible;15 h/wk for 8 wks preoperatively, 48 wks postoperatively.
2016, 2013 Oraee-Yazdani S.^133^, *N* = 8. Intrathecal injection, autologous BM-MSCs, and SCs	(Pre) to continue the same rehabilitation program for 6 mo to exclude dependency of any improvement to rehabilitation (Post) a regular rehabilitation program
2014 El-Kheir WA.^134^, Phase I–II, *N* = 70. Intrathecal injection, autologous BM-derived cells	PT programs; 1–2 h, 3 /wk. (Pre) no details were provided, (Post) started at 2–3 POD. Mat and transfer activities, self-ROM, strengthening, ambulation, upright posture on a tilt table, and cardiopulmonary training (same description with Kishk NA.^135^)
2014 Mendonça MV.^136^, *N* = 14. Intramedullary injection, BM-MSCs	Detail not described; started at 7 POD, for 6 mo, 4 h/d in first 2 mo, and 2 h/d thereafter, 5 /wk
2013 Larson CA.^15^, *N* = 13. Olfactory mucosa grating with scar removal	Intense outpatient PT; 3 h sessions, 3–5 /wk, for 4.6 mo (at least 3 mo) (i) pre-gait (weight-bearing, posture, balance, crawling, and standing) and/or gait training (BWSTT and overground gait), (ii) intense therapeutic exercise (repetitive neuromuscular facilitation, mat mobility, strengthening and endurance, whole-body vibration, biofeedback, virtual gaming, and/or musculoskeletal interventions), and (iii) FES cycling or static/dynamic standing frame activities
2013 Dai G.^137^, Phase I–II, *N* = 40. Intramedullary injection, autologous BM-MSCs	(Pre) to receive formal rehabilitation during the observation period to exclude the effect of rehabilitation (Post) detail not described
2013 Derakhshanrad N.^138^, Phase I, *N* = 12. Sural nerve in autologous fibrin coagulum grafting in the syrinx	(Pre) a standard rehabilitation at least 6 mo (Post) a wheelchair transfer at 48 h after Tp, and post-rehabilitation resumed
2013 Tabakow P.^98^, *N* = 6. Intramedullary injection, autologous OECs	ROM (60 min), locomotor training including treadmill (180 min), sensory training (60 min); 4–5 h/day, 3–5 d/wk for 3 mo preoperatively, 24 mo postoperatively.
2010 Kishk NA.^135^, *N* = 64. Monthly intrathecal injection, 6 mo, BM-MSCs	General rehabilitation: mat and transfer activities, self-ROM, strengthening, ambulation, tilt table, and cardiopulmonary training; 3 /wk, for 6 mo.
2008, 2011 Seberi H.^99 139^, *N* = 33. Intramedullary injection, SCs	Physical exercise, FES, ultrasonic diathermy and infrared; 3 h/day, 3 d/wk for 6 mo preoperatively, 12 mo postoperatively
2006, 2010 Lima C.,^14,140^, Phase I–II, *N* = 20. Olfactory mucosa grating with scar removal	Passive assisted ROM and strengthening; 2 h, functional training for balance, posture, standing, and transfers; 2–3 h, pre-gait, and gait activities. BWSTT, Lokomat®, BIONT which is an assisted overground walking training, with loading on hips, knees, and feet to promote sensorimotor biofeedback; 2–3 h. 31.8 h/wk, for 34.7 wks preoperatively, and 32.7 h/wk for 92 wks postoperatively
Jarocha D.^91^/Moviglia G.A.^13^/Cheng H.^141^/Goni V.G.^142^/Chernykh E.R.,^143^ Huang H.^92^/Cheng L.^145^	An intensive neurorehabilitation for 4 wks/Vojta and Bobath neurorehabilitation program/A functional recovery and urinary retention training/A standardized physical rehabilitation program/A regular rehabilitation/A rehabilitation for 6 mo.
Zhu H.^97^*, Yao L.^131^*, Wang S.^144^	No rehabilitation, *: no rehabilitation at one out of two sites
Thompson F.J.^146^, Wirth ED3rd.^147^, Feron F.^148^, Mackay-Sim A.^149^, Cristante A.F.^150^, Ra J.C.^151^, Wu J.^152^, Frolov A.A.^153^, Rao Y.^154^, Al-Zoubi A.,^155^ Vaquero J.^96,156,157^, Curtis E.^94^, Levi A.D.^95,158^	No description about rehabilitation

*AIS* ASIA impairment scale, *BM* bone marrow, *BWSTT* body weight-supported treadmill training, *FES* functional electrical stimulation, *MSC* mesenchymal stem cell, *OEC* olfactory ensheathing cell, *POD* post-operative day, *PT* physiotherapy, *ROM* range of motion, *Tp* transplantation, *SC* Schwann cell.

Since stem cell therapy has fewer effects on chronic SCI, combining rehabilitation attracts wide attention with its feasibility over the success in preclinical studies. Many research groups have implemented specific combined rehabilitative approaches. Furthermore, Huang et al.^92^ reported that the quality and quantity of rehabilitation influenced the long-term outcome in patients who underwent OEC transplantation, although they did not investigate the threshold. To our knowledge, this is the only study investigating the relationship between functional recovery and the characteristics of rehabilitation after stem cell therapy.

Rehabilitative interventions are often applied both before and after stem cell treatment in clinical studies involving patients with chronic SCI. We named the former “pre-rehabilitation” and the latter “post-rehabilitation”. Usually, only conventional rehabilitative methods are applied as pre-rehabilitation. Pre-rehabilitation can be categorized as mobilization of disuse-provoked impairments such as weakened and contracted muscles, homogenization of the status of patients before stem cell treatment, and identification of responsiveness to rehabilitation, which is far feasible than stem cell therapies. It is noteworthy that a study even excluded cases that showed a possibility of recovery with rehabilitation^93^.

## Strategies of regenerative rehabilitation applied in the clinical studies

Rehabilitative strategies appeared in regenerative studies can be characterized into six large groups:

1. The majority of groups seemed to apply only conventional rehabilitation. No rehabilitative protocol was provided in most cases^94–96^.
2. Intensive rehabilitation with the conventional method was also frequently applied. A massive dose of rehabilitation of >4–6 h per day was applied for a remarkably long duration exceeding 24 weeks up to 1 year^97^.
3. Several researchers chose a traditional, but specific, rehabilitative approach to maximize the effect of stem cell therapy. The principles of these approaches include facilitation of physical exercise^98,99^, early induction of training for essential motion including gait and ADL^14,15^, sensory training^14,15,98^, and the facilitation method^13^.
4. It was not rare for advanced rehabilitation, including FES^14,93,99^, NMES^100^, Upper body circuit resistance training^101^, BWSTT^14,15,99^, and RAGT^14^, to be implemented.
5. Physical treatments including electrical field, ultrasound, and temperature gradients were also applied to induce mechanotransductive effects^8,15,99^.
6. Home-based aerobic and strengthening program using dumbbell, resistance band, and upper limb ergometer^101^.

Although it is difficult to know the exercise load in home-based training, Maher et al.^101^ utilized the talk-test to determine an intensity that made speaking uncomfortable in maintaining the appropriate intensity level. We suggest that the second, third, fourth, fifth, and sixth groups are regenerative rehabilitation in a narrow sense, whereas the first group is combined rehabilitation, i.e., regenerative rehabilitation in a broad, but not a narrow, sense.

According to the mechanism revealed in preclinical and clinical studies, the roles of regenerative rehabilitation can be categorized as (i) conditioning/reconditioning, (ii) functional training, and (iii) physical exercise. Conditioning and reconditioning mainly target physical problems such as contracture, muscle atrophy, and cardiopulmonary deconditioning, which are derived from disuse due to SCI sequelae. They are even more important in the chronic phase because of the long deconditioning period^2,57,102^. Functional training will be the core element of regenerative rehabilitation. Although researchers have elucidated various micro-mechanisms via which SCI rehabilitation, in general, promotes functional recovery at the molecular, synaptic, local circuit, tract, and even cortical levels, the number of evidence showing interaction with residual tissue or transplanted cells is few in the field of regenerative rehabilitation. It is remarkable there has been no study investigating the effect brought about by rehabilitation regarding forelimb-hand function. Physical exercise is crucial to upregulate neurotrophic factors. These factors have remarkable effects including neuroprotection, especially in the acute phase, modification of proliferation of transplanted cells and their differentiation into neurons and oligodendrocytes, and induction of host neural plasticity^12,49,67^. Thus, physical exercise acts like medication. However, it remains to be elucidated to what degree the functional training and the physical exercise are distinguishable; whereas these two approaches possess a different character, there is still some overlapping. Further investigations are needed.

## Perspective for regenerative rehabilitation for SCI

In the field of stem cell regenerative medicine, the role of rehabilitation is becoming increasingly important, with preclinical studies delineating the molecular mechanisms and effects. Studies of combinatorial treatment have reported synergistic effects^11,12,64^. Although stem cell therapy seems to induce only limited recovery, the additive effect of regenerative rehabilitation may be key to realizing significant motor and functional recovery following chronic SCI. However, it seems difficult for combinatorial treatments incorporating stem cell therapy and rehabilitation to outperform each individual treatment, particularly in the chronic phase^10,11,15^. Therefore, it is crucial to optimize regenerative rehabilitation based on the mechanisms to maximize the effects of stem cell therapy. In addition, further investigations are needed to discover a way of enhancing recovery.

To date, researchers are pointing out the therapeutic potential to combine stem cell therapy and rehabilitation with pharmacological treatments as represented by neurotrophic factors and reagents against axonal growth inhibitor^1,2^. Combination treatment with emerging novel technologies from the fields of bioengineering or molecular biology has evolvability either. For example, neuromodulation with multimodal therapies including spinal epidural stimulation, brain–computer interface, neuroprosthetics, and pharmacological intervention is expected to restore functional movement upon inducing plasticity of spared circuits and residual projection^81^. On the other hand, in vivo reprogramming technology, which enables the generation of new neurons from non-neuronal cells via reprogramming, was recently developed, which is proposed as a novel regenerative strategy^103^. While no study has combined these groundbreaking technologies with stem cell therapy or rehabilitation, to the best of our knowledge, we believe that such extended regenerative rehabilitative strategies will further broaden the therapeutic potential of SCI.

## Supplementary information

Supplementary Information

## Data Availability

Data sharing is not applicable to this article as no datasets were generated or analyzed during the current study.
