# Supplementary material for: Current progress of rehabilitative strategies in stem cell therapy for spinal cord injury: a review
Source: NPJ Regen Med. 2021 Nov 25;6:81. doi: 10.1038/s41536-021-00191-7 (PMC8616941; doi:10.1038/s41536-021-00191-7)
Supplement: Supplementary file 1 — Supplementary Information [file 41536_2021_191_MOESM1_ESM.pdf]

Supplementary Table 1.

SCI rehabilitation in preclinical studies on stem cell therapies, a full version of Table 1

Study information	Rehabilitation	Specific effect of regenerative rehabilitation
2021 Sun WM. [70]<br>-Animal; rhesus monkey, male, N = 4<br>-Phase; super-acute (immediately after SCI)<br>-SCI; hemisection (T8)<br>-Graft; sural nerve graft combined with a long-term acidic fibroblast growth factor infusion<br>-Transplantation; injection to bridge lesion	Regular stretching, standing with supportive monkey chair, positioning for flexibility and reducing muscle spasm, monkey-chair supported treadmill ambulation<br>-Initiation and period; not described<br>-Duration; two 20-min sessions / day<br>-Frequency; 5 days / week	-Untrained condition was not investigated.
2021 Prager J. [71]<br>-Animal; Wistar rats, male, N = 23<br>-Phase; super-acute (immediately after SCI)<br>-SCI; dorsal column crush corticospinal tract injury, (C3)<br>-Graft; canine olfactory mucosal olfactory ensheathing cells to express chondroitinase ABC<br>-Transplantation; injection at: 2 sites cranial and caudal to the lesion	Forepaw reaching rehabilitation<br>-Initiation; 1 week after transplantation<br>-Period; 8 weeks<br>-Duration; 1hour / day<br>-Frequency; 5 days / week	-Untrained condition was not investigated.
2020 Younsi A. [67]<br>-Animal; Wister rat, female, N = 70<br>-Phase; subacute (10 days post SCI)<br>-SCI; moderate contusion, C6<br>-Graft; neural precursor cells<br>-Transplantation; injection at 4 sites (bilaterally, rostral and caudal)	Quadrupedal treadmill training<br>-Initiation; 48 hours after transplantation<br>-Period; 6 weeks<br>-Duration; 20 min / day	-Graft survival, and differentiation into neurons and oligodendrocytes increased.<br>-Myelination, regeneration of descending tracts and tissue sparing improved.<br>-Better functional recovery with synergistic effect.
2020 Dugan EA [74]<br>-Animal; Sprague-Dawley rat, male, N = 48<br>-Phase; late subacute (28 DPI)<br>-SCI; clip contusion, T6-7<br>-Graft; GABAergic neural progenitor cells<br>-Transplantation; injection into lumbar enlargement (different site from injury)	Quadrupedal 8 degree incline treadmill training in ramping protocol<br>-Initiation; 5 days after SCI or 5 weeks after SCI<br>-Period; 4 weeks, 5 days / week<br>-Duration; 20 min / day	-Enhancement of neuropathic pain reduction as assessed by allodynia, hyperalgesia, and ongoing pain.<br>-Improvement of pro-inflammatory markers and spinal pathology in lumbar dorsal horn<br>-Restoration of GABAergic neuronal and process density<br>-BDNF expression
2020 Massoto TB. [66]<br>-Animal; C57 Black 6, female, N = 40<br>-Phase; subacute (7 DPI)<br>-SCI; clip contusion, T9<br>-Graft; bone marrow cells<br>-Transplantation; injection into the lesion epicenter	Quadrupedal treadmill training<br>-Initiation; 14 days after SCI<br>-Period; 8 weeks, 3 days / week<br>-Duration; 10 min / day	-Significantly better functional result<br>-A larger spared white matter and a higher number of preserved myelinated fibers<br>-Few microcavitations and degenerating nerve fibers<br>-Higher neurotrophin-4 expression.
2018 Thornton MA. [104]<br>-Animal; Sprague-Dawley rats, female, N = 10<br>-Phase; super-acute (just after SCI)<br>-SCI; thoracic cord transection, (T6-7)<br>-Graft; olfactory ensheathing cells<br>-Transplantation; injection at 2 sites rostral and caudal to the lesion	Climb training combined with 40 Hz epidural stimulation during training at a 95 % of motor threshold<br>-Initiation; 1 month after transplantation<br>-Period; 6 month<br>-Duration; 20 min / day<br>-Frequency; 3 times / week	-No control group regarding training and electrical stimulation evaluated.
2018 Tashiro S. [10]<br>-Animal; C57 Black 6j mouse, female, N = 45<br>-Phase; chronic (49 days post SCI)<br>-SCI; severe contusion, T9<br>-Graft; murine E14 fetal neural stem/progenitor cells<br>-Transplantation; injection at 2 sites (rostral and caudal)	Bipedal treadmill training<br>-Pretraining: 1 week<br>-Initiation: 3 days after transplantation<br>-Period: 8 weeks, 5 days / week<br>-Duration: 20 min / day	-Amelioration of thermal allodynia and coarse touch-pressure hyperalgesia<br>-Restoration in GABAergic activity in the posterior horn of lumbar spinal cord.
2017 Theisen CC.[22]<br>-Animal; Sprague-Dawley rat, female, N = 45<br>-Phase; late subacute-chronic (transplantation at 42 DPI, training started at 35 DPI)<br>-SCI; complete transection, T12<br>-Graft; harvested Tibial nerve to graft the injury site	Cycling exercise<br>-Initiation: Acute group, 5 days post SCI; Delayed group, 35 days post SCI<br>-Period: 10 weeks for acute group, 6 weeks for delayed group<br>-Duration: 30 min / day	-Enhancement of spinal axons regeneration into peripheral nerve grafts after exercise<br>- No remarkable benefit with a longer pre-graft exercise period
2016 Nicola FC. [61]<br>-Animal; Wistar rats, N = 54<br>-Phase; super-acute (immediately after SCI)<br>-SCI; moderate contusion, T9<br>-Graft; human exfoliated deciduous teeth stem cell, without immunosuppression<br>-Transplantation; injection at the epicenter	Quadrupedal treadmill training<br>-Pretraining: 1 week<br>-Initiation: 3 days after transplantation<br>-Period: 6 weeks, 5 days / week<br>-Duration: 10 min / day	-No significant additional effect with combination of training

Study information	Rehabilitation	Specific effect of regenerative rehabilitation
2016 Tashiro S. [11]<br>-Animal; Black 6j mouse, female, N = 80<br>-Phase; chronic (49 days post SCI)<br>-SCI; severe contusion, T9<br>-Graft; murine E14 fetal neural stem/progenitor cells<br>-Transplantation; injection at 2 sites (rostral and caudal)	Bipedal treadmill training<br>-Pretraining: 1 week<br>-Initiation: 3 days after transplantation<br>-Period: 8 weeks, 5 days / week<br>-Duration: 20 min / day	-Facilitation neuronal differentiation of transplanted cells<br>-Enhancement of neurogenesis in lumbar enlargement.<br>-Additive effect of enhanced serotoninergic activity from transplantation and restoration of GABAergic activity from training
2016 Sachdeva R.[63]<br>-Animal; Sprague-Dawley rat, female, N = 45<br>-Phase; super-acute (just after the SCI)<br>-SCI; complete transection, T12<br>-Graft and Transplantation; harvested Tibial nerve grafted in the injury site	Cycling exercise, 45 rotations per minute<br>-Initiation: 5 days after SCI<br>-Period: 4 weeks, 5 days / week<br>-Duration: 30 min / day	-Exercise enhanced a propriospinal neuronal regeneration in more distant regions (5–20 mm from the lesion), accompanying mRNA expressions of GAP43, β-actin and Neuritin<br>-No exercise-associated increase in regeneration regarding sensory / DRG neurons.
2015 Dugan EA.[105]<br>-Animal; Sprague-Dawley rat, female, N = 64<br>-Phase; super-acute (immediately after SCI)<br>-SCI; transection, T10<br>-Graft; neural / glial restricted precursor cells<br>-Transplantation; injection into lesion cavity<br>-Medication; serotonin receptor agonist, quipazine, treatment from 2 weeks after SCI	Quadrupedal cycling passive exercise<br>-Initiation: 5 days after transplantation<br>-Period: up to 12 weeks, 5 days / week<br>-Duration: 60 min / day (two 30-min sessions with a 10-min rest inbetween)	-Weight-supported gait was only achieved in the combination treatment group under quipazine condition<br>-no regeneration of descending serotonergic projections into and through the lesion cavity
2014 Hwang DH.[12]<br>-Animal; Sprague-Dawley rat, female, N = 184<br>-Phase; sub-acute (7 DPI)<br>-SCI; moderate contusion, T9<br>-Graft; neural progenitor cells<br>-Transplantation; injection at 4 sites (bilaterally, rostral and caudal)	Quadrupedal treadmill training<br>-Pretraining: 1 week<br>-Initiation: 3 days after transplantation<br>-Period: 8 weeks, 6 days / week<br>-Duration: 60 min / day	-Improvement in graft cell survival and differentiation into neurons and oligodendrocyte, with unchanged astrocyte differentiation and less undifferentiated cells.<br>-Attenuation in cellular stresses from reactive nitrogen and oxygen via IGF-1 signaling.<br>-Up-regulation of BDNF, GDNF, and NT-3.<br>-Increase in spared tissue, myelinated area, and 5-HT positive fiber.
2013 Sun T. [65]<br>-Animal; Sprague-Dawley rat, female, N = 40<br>-Phase; sub-acute (14 DPI)<br>-SCI; moderate contusion, T10<br>-Graft; olfactory ensheathing cells and Schwann cells<br>-Transplantation; injection at 2 sites (rostral and caudal, 4 different depth)	Bipedal treadmill training<br>-Initiation: 7 days after transplantation<br>-Period: 10 weeks<br>-Duration: 20 ± 10 min / day	-Enhancement of increased Serotonin activity at lumbar enlargement.
2013 Zurita M. (Observation study) [159]<br>-Animal; Minipig, female, N = 4<br>-Phase; chronic (3 months post SCI)<br>-SCI; clip contusion, L2-3<br>-Graft; autologous bone marrow stromal cell<br>-Transplantation; Perilesional intrathecal administration	Quadrupedal training<br>-Initiation: 1 week after SCI<br>-Period; throughout study period, several months	-Clinical improvement starting at 2 months<br>-Restoration of somatosensory-evoked potentials<br>-Improvement in histological spinal cord regeneration
2012 Penha EM. (Observation study) [160]<br>-Animal; Felis catus, male, N = 1<br>-Phase; super-acute (immediately after SCI)<br>-SCI; compressive L1-L5 fracture<br>-Graft; autologous mesenchymal stem cells<br>-Transplantation; injection	Comprehensive method including functional electrical stimulation<br>-Initiation: when hematological, renal and hepatic indices recovered<br>-Duration and Period; Intensive: 2 hours x 2 times / day for 2 months<br>Reduced: 1 hours / day for 2 months	-First steps, three-minute weight-bearing, and intestine and urinary bladder partial reestablishment were observed 75 days post-surgery<br>(Only behavioral results presented)
2011 Takeoka A. [64]<br>-Animal; Wistar Hannover rat, female, N = 41<br>-Phase; super-acute (immediately after SCI)<br>-SCI; complete transection, T9<br>-Graft; olfactory ensheathing glia<br>-Transplantation; injection at rostral and caudal side	Bipedal step and treadmill gait graining<br>-Initiation: 2 weeks after SCI and transplantation<br>-Period: 7.5 months<br>-Duration: 20 min	-Enhancement of some stepping measures<br>-Transplantation alone promoted reorganization of lumbosacral locomotor networks and restoration of motor-evoked-potentials
2008 Kubasak MD. [62]<br>-Animal; Wistar Hannover rat, female, N = 38<br>-Phase; super-acute (immediately after SCI)<br>-SCI; complete transection, T9<br>-Graft; olfactory bulb-derived olfactory ensheathing glia<br>-Transplantation; injection at each 4 sites in rostral and caudal stump	Bipedal treadmill step<br>-Pretraining: acclimated to device for 2 weeks before SCI<br>-Period: 6 months<br>-Duration: 5 min in first week, and increased by 5 min each week for the first month. 20 min / day, thereafter.	-Improvement in stepping abilities between the 4- and 7-month evaluations with increased number of plantar stepping.<br>-No comparison between trained and untrained groups in histological assessments.

Study information	Rehabilitation	Specific effect of regenerative rehabilitation
2008 Carvalho KAT.[106]<br>-Animal; Wistar rat, male, N = 48<br>-Phase; acute (48 hours after SCI)<br>-SCI; mild contusion, T9-10<br>-Graft; bone marrow stem cell (CD45+/CD34-)<br>-Transplantation; injection	Swimming training supporting an overload of 3% of body weight<br>-Period 6 weeks<br>-Duration; 60 min<br>-Frequency; 6 times / week	-Combination resulted in significant functional improvement against transplanted-sedentary rat<br>(Motor assessment only)
2006 Yoshihara H.[60]<br>-Animal; Sprague-Dawley rat, female, N = 26<br>-Phase; subacute (9 days after SCI)<br>-SCI; mild contusion, T9-10<br>-Graft; bone marrow stromal cells<br>-Transplantation; injection at lesion epicenter, and the rostral and caudal edges of lesion	Passive hindlimb exercise on motorized bicycle, 45 rotations per minute<br>-Initiation: 2-3DPI<br>-Period and Frequency: 3 days / week for 3 months<br>-Duration: 30-min-exercise, 10-min-rest, 30-min-exercise	-No difference in cell survival, evidence of axonal growth into grafts, phenotype, and lesion/ transplant size.
2006 Lynskey JV.[107]<br>-Animal; Sprague-Dawley rat, female, N = 47 out of 84 are trained<br>-Phase; subacute (2 weeks post SCI)<br>-SCI; over-hemisection, C5/6<br>-Graft; Fetal spinal cord tissue<br>-Transplantation; 5–6 pieces of tissue with Gelfoam placed over the lesion	Direct skilled target reaching<br>-Initiation; 4 weeks after transplantation<br>-Period; 5 days<br>-Duration; 5-10 min	-Untrained condition was not investigated.<br>-Rats treated with transplantation and NT3 administration exerted greatest recovery.
2005 Keyvan-Fouladi N.[72]<br>-Animal; Albino Swiss strain rat, female, N = 46<br>-Phase; chronic (8 weeks post SCI)<br>-SCI; radiofrequency lesion at corticospinal tract and adjacent grey matter, C1/2<br>-Graft; olfactory ensheathing cells<br>-Transplantation; injection at 1 site to bridge the lesion	Direct forepaw reaching<br>-Initiation; 3 days after transplantation<br>-Period; 8 weeks<br>-Duration; 50 retrieval / limb<br>-Frequency; 3 times / week	-Untrained condition was not investigated.
2005 Ruitenberg MJ.[108]<br>-Animal; Fischer 344 rat, female, N = 29<br>-Phase; chronic (8 weeks post SCI)<br>-SCI; cervical-dorsal hemisection (C4)<br>-Graft; olfactory ensheathing glia (OEG)<br>-Transplantation; injection at2 sites	Direct forepaw reaching<br>-Initiation; 2 weeks after transplantation<br>-Period; 10 weeks<br>-Frequency; 3 times / week	-Untrained condition was not investigated.<br>-OEG-NT3 treated rats showed the highest recovery.
2003 Keyvan-Fouladi N.[109]<br>-Animal; Albino Swiss strain rat, female, N = 54<br>-Phase; chronic (8 weeks post SCI)<br>-SCI; cervical-dorsal hemisection (level not described)<br>-Graft; olfactory ensheathing cells<br>-Transplantation; injection at 1 site to bridge the lesion	Direct forepaw reaching<br>-Initiation; 3 days after transplantation<br>-Period; 8 weeks<br>-Duration; 50 retrieval / limb<br>-Frequency; 3 times / week	-Untrained condition was not investigated.

Abbreviations

DPI: Days post injury, SCI: Spinal cord injury

Supplementary Table 2.

SCI rehabilitation in clinical studies on stem cell therapies involving acute/subacute patients, a full version of Table 2

Study information	Transplantation details	Rehabilitation
2020 Chen W.[100]<br>Xinquao hospital affiliated with the Army Medical University, Chongqing, China<br>Phase of clinical trial: I, N = 7	-Phase: acute<br>-Graft: autologous bone marrow mononuclear cells loaded into NeuroRegen scaffolds which is collagen scaffold containing multiple nerve regeneration molecules<br>-Procedure: implanted into the cleaned site	6 month of standard rehabilitation including routine care, hyperbaric oxygen therapy, neurotrophic therapy, acupuncture, neuromuscular electrical stimulation therapy, upper limb muscle strength training, self-care training, etc.
2020 Sharma A.[110]<br>NeuroGen Brain and Spine Institute, Navi Mumbai, India<br>Open-label study, N = 180	-Phase: mixture (subacute and chronic)<br>-Graft: autologous bone marrow mononuclear cells<br>-Procedure: intrathecal injection	Neurorehabilitation, including physiotherapy, occupational therapy, psychological interventions, and aquatic therapy. A home program under the supervision of a professional was recommended.
2018 Xiao Z.[122]<br>Institute of Genetics and Developmental Biology, Chinese Academy of Sciences, Beijing, China<br>Phase of clinical trial: I, N = 2	-Phase: acute<br>-Graft: collagen scaffolds with human umbilical cord mesenchymal stem cells<br>-Procedure: transplanted into the injury site	A standard rehabilitation
2017 Anderson KD.[111]<br>Miami Miller School of Medicine, Florida, US<br>Phase of clinical trial: I, N = 6<br>*A study to develop a rehabilitation home program was derived from this trial.[101]	-Phase: subacute<br>-Graft: autologous human Schwann cells<br>-Procedure: injected into the epicenter of the spinal lesion	Inpatient standard medical rehabilitation<br>(Pre-transplantation)<br>during the 3–5 weeks that required for cell processing<br>(Post-transplantation)<br>three hours each day, five days each week, for 6.6 ± 2.1 weeks
2016 Bansal H.[121]<br>Mother Cell, Anupam Hospital, Rudrapur, Uttarakhand & RegennMed Research &Therapeutics, New Delhi, India<br>Phase of clinical trial: I, I/II, N = 7 for subacute, 3 for chronic	-Phase: mixture (subacute and chronic)<br>-Graft: autologous bone marrow stem cells<br>-Procedure: repetitive intrathecal injection every 4 weeks 3 times	A standard rehabilitation
2016 Satti HS.[119]<br>Quaid-i-Azam University, Islamabad, Pakistan<br>Phase of clinical trial: I, I, N = 6 for subacute, 6 for chronic	-Phase: mixture (subacute and chronic)<br>-Graft: autologous mesenchymal stromal cell<br>-Procedure: intrathecal administration	A rehabilitation program (not specified)
2016 Hur JW.[120]<br>Korea University College of Medicine, Korea University Anam Hospital, Seoul, South Korea<br>Phase of clinical trial: I, N = 3 for late subacute, 11 for chronic.	-Phase: mixture (chronic and subacute)<br>-Graft: adipose-derived mesenchymal stem cells<br>-Procedure: repetitive intrathecal injection every 4 weeks 3 times	A standard rehabilitation
2015 Chhabra HS.[118]<br>Indian Spinal Injuries Center, New Delhi, India.<br>Phase of clinical trial: I, not described, N = 14, Control = 7	-Phase: acute<br>-Graft: autologous bone marrow transplantation<br>-Procedure: injection into six separate positions surrounding the lesion site (two above, two below and two at the lesion site) with the injection depth of 5mm	A standard rehabilitation
2015 Shin JC.[116]<br>Yonsei University College of Medicine, Seoul, South Korea<br>Phase of clinical trial: I/IIa N = 18 for subacute, 1 for chronic, Control N =15 for subacute	-Phase: mixture (subacute and chronic)<br>-Graft: human neural stem/progenitor cells-derived from the fetal telencephalon<br>-Procedure: injected into the spinal cord using a 23-gauge needle	A standard rehabilitation
2013 Liu J.[112]<br>General Hospital of the Air Force, Beijing, China<br>Phase of clinical trial: not described, N = 22	-Phase: mixture (subacute and chronic)<br>-Graft: umbilical cord mesenchymal stem cells<br>-Procedure: intrathecal injection	A systemic individualized physical therapy
2012 Karamouzian S.[117]<br>Kerman University of Medical Sciences, Bahonar Hospital, Kerman, Iran.<br>Phase of clinical trial: not described, N = 11 (N = 20 for Control)	-Phase: subacute<br>-Graft: autologous bone marrow cell<br>-Procedure: intrathecal injection	A conventional rehabilitation
2012, 2008 Saito F.[126,127]<br>Kansai Medical University, Osaka, Japan<br>Phase of clinical trial: not described, N = 3 (N = 2 for Control)	-Phase: subacute<br>-Graft: autologous bone marrow stromal cells<br>-Procedure: intrathecal injection	No description about rehabilitation

Study information	Transplantation details	Rehabilitation
2012 Lammertse DP.,[129] 2010 Jones LAT.,[128] 2005 Knoller N.[115]<br>Proneuron Biotechnologies, Englewood, Colorado,<br>Multi-center (6 institute): Chaim Sheba Medical Center, Israel, and Craig Hospital, Mount Sinai School of Medicine, Kessler Medical Rehabilitation Research & University of Medicine and Dentistry Newark, Shriners Hospital Philadelphia, Shepherd Center Atlanta, US<br>Phase: II, N = 50 for treatment, 25 for control<br>2) Phase of clinical trial: I, N = 8,<br>3) Phase of clinical trial: II, N = 26 for treatment, 17 for control	-Phase: acute<br>-Graft: autologous macrophages<br>-Procedure: injected in the caudal boarder of lesion	1, 2) No description about rehabilitation<br>3) A standard rehabilitation
2011 Attar A.[124]<br>Ankara University, Ankara, Turkey<br>Phase of clinical trial: I, N = 4	-Phase; subacute<br>-Graft: autologous bone marrow mononuclear cells<br>-Procedure: intramedullary injection	Transferred to rehabilitation clinic at 15 post-operative day. Rehabilitation duration not described.
2009 Kumar AA.[125]<br>Lifeline Institute of Regenerative Medicine, Chennai, India<br>Phase of clinical trial: I/II, N = 297	-Phase: acute<br>-Graft: autologous bone marrow derived mononuclear cell<br>-Procedure: intrathecal injection	No description about rehabilitation
2009 Pal R.[123]<br>Stempeutics Research Private Ltd, Bangalore, India<br>Phase of clinical trial: not described, Acute/Subacute: N = 20, Chronic: N = 10	-Phase: mixture (acute/subacute and chronic)<br>-Graft: bone marrow-derived mesenchymal stromal cells<br>-Procedure: intrathecal injection, once a week, 2-3 times.	No description about rehabilitation
2007 Yoon SH.[113]<br>Inha Neural Repair Center, Korea<br>Phase: I / II, Acute (< 14 DPI): N = 17, Subacute (>= 14 DPI, < 8 weeks): N = 6, Subacute-Chronic (>= 8 weeks) : N = 12.	-Phase: mixture (acute to chronic)<br>-Graft: autologous bone marrow cell<br>-Procedure: injected into six separate positions surrounding the lesion site	An active rehabilitation
2003 Ravinovich SS.[114]<br>Nobosibirsk State Medical Academy<br>Phase of clinical trial: not described, N = 15	-Phase: mixture (subacute to chronic)<br>-Graft: fatal nervous and hemopoietic tissues<br>-Procedure: subarachnoidal implantation. Operative partial disruption of connective tissue cyst combined with olfactory ensheathing cell implantation in 11 out of 15 patients.	A standard rehabilitation before transplantation.<br>No information about rehabilitation post-treatment.

<u>Abbreviations</u>

DPI: Days post injury, SCI: Spinal cord injury

Supplementary Table 3

SCI rehabilitation in clinical studies on stem cell therapies for chronic patients, a full version of Table 3

Study information	Transplantation details	Rehabilitation
2021 Gant KL.[130]<br>University of Miami<br>Phase I, N=8 (treatment, N = 4; control, N = 4)	-Graft: autologous human Schwann cell<br>-Procedure: injection with cavity-filling volume approach	Conditioning of lower extremities: FES and cycle ergometer; twice a week.<br>Body-weight supported locomotor training with treadmill-based robotic gait for AIS A or B participants, or overground locomotor skill training for AIS C participants; biweekly.<br>Upper extremity circuit resistance training; three times a week on non-consecutive days.
2018, 2019 Levi AD.[95,158]<br>Miami Miller School of Medicine<br>Phase II, N = 12	-Graft: human CNS stem cell<br>-Procedure: pelilesional intramedullary injections	No description about rehabilitation
2018 Curtis E.[94]<br>University of California, San Diego<br>Phase I, N = 4	-Graft: human spinal cord-derived NSCs<br>-Procedure: six midline bilateral stereotactic injections	No detailed description about rehabilitation<br>Patients did not receive any additional rehabilitation beyond their routine outpatient rehabilitative therapy
2016, 2017, 2018 Vaquero J.[96,156,157]<br>University Hospital Puerta de Hierro-Majadahonda, Madrid<br>Phase II, N = 11	-Graft: autologous mesenchymal stromal cells<br>-Procedure: intrathecal administration	No description about rehabilitation
2016 Zhu H.[97], Yao L.[131]<br>Kunming General Hospital of Chengdu Military Command, Yunnan<br>Chinese University of Hong Kong<br>Phase I-II, N = 28	-Graft: umbilical cord blood-derived mononuclear cell<br>-Procedure: rostral and caudal intramedullary injections	Intensive locomotor training only at Kumming but not at Hong Kong<br>Initiated 14 days after surgery<br>6 hours / day, 6 days / week for 3–6 months.<br>3 hours in the morning and 3 hours in the afternoon
2016 Oh SK.[93]<br>Asan Medical Center, University of Ulsan College of Medicine, Seoul<br>Phase III, N = 16	-Graft: autologous mesenchymal stem cells<br>-Procedure: subdural injection	(Pre-transplantation)<br>Included only when no improvements with 3 months rehabilitation<br>(Post-transplantation)<br>Initiated one week after surgery<br>A standardized rehabilitation 6 days per week for a total of 4 weeks, which includes physical therapy twice a day, lying on the tilting table for 30 minutes, and mat exercises for 30 minutes. Mat exercises included active assistive range of motion for upper and lower extremities, functional activities such as rolling and sitting. FES was applied on the wrist extensor and quadriceps
2016 Wang S.[144]<br>Wenzhou Medical University, Wenzhou, Zhejiang, China<br>Phase I-II, N = 8, 4 for control	-Graft: autologous olfactory lamina propria<br>-Procedure: transplantation into the cavity with posterior midline myelotomy or scarlotomy	No rehabilitation: physical exercises or rehabilitation programs had not been initiated after the patients were discharged from the hospital because of financial difficulties and other major social disadvantages
2016 Iwatsuki K.[132]<br>Osaka University Medical School, Suita, Osaka, Japan<br>Phase of clinical trial: not described, N = 8	-Graft: olfactory mucosa<br>-Procedure: pieces of olfactory mucosa were grafted into lesion site together with the removal of scar tissue	(Pre-transplantation)<br>15 hours / week for a duration of 8 weeks<br>(Post-transplantation)<br>15 hours / week for a duration of 48 weeks<br>(Rehabilitation)<br>A standard physical therapy strategies to encourage motor function at and below the lesion, enabling walking training as soon as possible
2016, 2013 Oraee-Yazdani S.[133]<br>Shahid Beheshti University of Medical Sciences, Teheran, Iran<br>Phase I study in 2016, N = 6, and Report in 2013, N = 8	-Graft: co-transplantation of autologous bone marrow mesenchymal stem cells and Schwann cells<br>-Procedure: intrathecal injection	(Pre-transplantation)<br>Let the participants to continue the same rehabilitation program they had received for 6 months before transplantation to exclude dependency of any improvement to the rehabilitation program.<br>(Post-transplantation)<br>Regular rehabilitation program after transplantation
2014 Mendonça MV.[136]<br>Hospital Espanhol, Salvador, Brazil<br>Phase I, N = 14	-Graft: bone marrow-derived mesenchymal stem cells<br>-Procedure: injected directly into the lesion	Initiated one week after the surgical procedure<br>Continued for 6 months: 5 times / week, for 4 hours / day during first 2 months; 2 hours / day in subsequent months
2014 Cheng H.[141]<br>General Hospital of Chinese people's Armed Police Forces, Beijing, China<br>Phase of clinical trial: not described, N = 10, 24 for control	-Graft: umbilical cord mesenchymal stem cells<br>-Procedure: CT guided injection at the junction of normal-injured area twice at 10-days interval	A functional recovery training and urinary retention training.

Study information	Transplantation details	Rehabilitation
2014 Cheng L.[145]<br>Beijing Rehabilitation Hospital<br>Phase of clinical trial: not described, N=7	-Graft: olfactory ensheathing cells and Schwann cells<br>-Procedure: injection in the dorsal midline, above and below the lesion.	6-month rehabilitation program, which detail is not provided.
2014 Al-Zoubi A.[155]<br>University of Illinois College of Medicine at Peoria, USA<br>Phase of clinical trial: not described, N=19	-Graft: leukapheresis-derived CD34+/CD133+ stem cells<br>-Procedure: directly transplanted into the lesion	No description about rehabilitation
2014 Goni VG.[142]<br>Post Graduate Institute of Medical Education and Research, Chandigarh, India<br>Phase not described, N=9	-Graft: olfactory mucosa and bone marrow stem cells<br>-Procedure: injection into the cavity after the scar removal	A standardized physical rehabilitation program
2014 Jarocha D.[91]<br>University Children's hospital, Cracow, Poland<br>Preliminary study for Children, N = 19	-Graft: bone marrow nucleated cells<br>-Procedure: intraspinal transplantation with resection of necrotic tissue, and intravenous	An intensive neurorehabilitation for 4 weeks
2013 Rao Y.[154]<br>Luoyang Orthopaedic–Traumatological Hospital, Luoyang, China<br>Phase of clinical trial: not described, N = 8	-Graft: autologous olfactory ensheathing cells<br>-Procedure: MR-guided injection into 6 sites at caudal border of the lesion	No description about rehabilitation
2013 Larson CA.[15]<br>Center for Spinal Cord Injury Recovery, Detroit, Michigan, US<br>Phase of clinical trial: not described, N = 7: transplantation, 6: control<br>*Study for combined treatment with intensive physiotherapy and transplantation	-Graft: olfactory mucosa<br>-Procedure: pieces of olfactory mucosa were grafted into lesion site together with the removal of scar tissue	Intense, outpatient, physiotherapy<br>Three hour sessions, 3–5 times per week for a minimum duration of three months (mean: 4.6 months)<br>Therapeutic activities and exercises as determined by the primary physical therapist (i) pre-gait (i.e. weight bearing in multiple positions, posture and balance training, crawling, and standing pre-gait activities) and/or gait training (i.e. BWSTT and over-ground gait training), (ii) intense therapeutic exercise (i.e. repetitive neuromuscular facilitation, mat mobility, strengthening and endurance exercises, whole body vibration, biofeedback, virtual gaming, and/or musculoskeletal interventions), and (iii) FES cycling or static/dynamic standing frame (glider) activities.
2013 Dai G.[137]<br>The Military General Hospital of PLA, Beijing<br>Phase: I-II. N = 20, 20 for control	-Graft: autologous bone marrow mesenchymal stem cells<br>-Procedure: injected to the area surrounding injury	To exclude the effect of rehabilitation exercises on neurological rehabilitation, all patients received formal rehabilitation exercises at the same hospital during the observation period
2013 Derakhshanrad N.[138]<br>Tehran University of Medical Sciences, Tehran, Iran.<br>Phase I, N = 12	-Graft: autologous peripheral nerve grafts<br>-Procedure: Sural nerve embedded in the autologous fibrin coagulum as one piece, and were placed in the syrinx.	(Pre-transplantation)<br>A standard rehabilitation at least 6 months<br>(Post-transplantation)<br>Wheel chair transfer at 48 hours after transplantation<br>post-rehabilitation resumed
2013 Tabakow P.[98]<br>Wroclaw Medical University, Wroclaw, Poland.<br>Phase I, N = 6	-Graft: autologous olfactory ensheathing cells<br>-Procedure: stereotactic intraspinal cell grafting	Duration: Pre, 3 months; Post, 24 months, 4–5 hours / day, 3–5 days / week<br>Range of motion, 60 minutes; locomotor training including treadmill, 180 minutes; sensory training, 60 minutes
2012 Frolov AA.[153]<br>NeuroVita Clinic of Interventional and Restorative Neurology and Therapy, Moscow, Russia.<br>Phase of clinical trial: not described, N = 20	-Graft: hematopoietic autologous stem cell<br>-Procedure: intrathecal injection	Pre-transplantation: several months<br>Post-transplantation: not described
2012 Wu J.[152]<br>Beijing Army General Hospital<br>Phase I, N = 6	-Graft: fetal olfactory ensheathing glia<br>-Procedure: injected to the area rostral and caudal to the injury	No description about rehabilitation
2012 Huang H.[92]<br>Beijing Rehabilitation Center<br>Phase of clinical trial: not described, N = 108	-Graft: olfactory ensheathing cells<br>-Procedure: injection	79 patients received sufficient rehabilitation, whereas 29 did not.<br>Motor and sensory functional recovery were significantly enhanced by sufficient rehabilitation.<br>Criteria for sufficient rehabilitation was not provided.
2011 Ra JC.[151]<br>Stem cell Research Center, RNL Bio Co, Ltd, Seoul, Korea<br>Phase I, N = 8	-Graft: adipose tissue-derived mesenchymal stem cells<br>-Procedure: intravenous injection	No description about rehabilitation

Study information	Transplantation details	Rehabilitation
2008, 2011 Seberi H.[99 139]<br>Tehran University of Medical Sciences/Tehran University<br>Phase of clinical trial: not described, N = 33	-Graft: Schwann cell acquired from autologous sural nerve<br>-Procedure: injected directly into the lesion	Pre: 6 months<br>Post: 12 months<br>3 hours / day, 3 days / week, and it continued throughout the study period (physical exercise, FES, ultrasonic diathermy and infrared).
2006, 2010 Lima C.[14,140]<br>Centro Hospitalar de Lisboa Ocidental, Lisbon, Portugal<br>Phase I-II, N=20	-Graft: olfactory mucosa<br>-Procedure: pieces of olfactory mucosa were grafted into lesion site together with the removal of scar tissue	preoperative rehabilitation (31.8 ± 6.8 hours / week, mean duration of 34.7 ± 30 weeks) and postoperative rehabilitation (32.7 ± 5.2 hours / week mean duration of 92 ±37.6 weeks)<br>Encouraging motor function at and below the lesion, enabling walking training as soon as possible<br>2 hours of passive, assisted range of motion and strengthening exercises; 2 to 3 hours of functional training for balance, posture, standing, and transfers; and 2 to 3 hours of pregait and gait activities. BWSTT, Lokomat®, BIONT which is an assisted overground walking training, with loading on the hips, knees, and feet to promote sensorimotor biofeedback.
2009 Cristante AF.[150]<br>Hospital das Clínicas, University of São Paulo School of Medicine, Brazil<br>Phase of clinical trial: not described, N = 29	-Graft: bone marrow stem cells<br>-Procedure: intra-arterial infusion	No description about rehabilitation
2007 Chernykh ER.[143]<br>Siberian Division of the Russian Academy of Medical Sciences. Russia<br>Phase of clinical trial: not described, N = 36	-Graft: autologous bone marrow cells<br>-Procedure: injected into the cyst cavity and intravenously	A regular rehabilitation
2006 Moviglia GA.[13]<br>Instituto Regina Mater, Buenos Aires, Argentina<br>Phase of clinical trial: not described, N = 2	-Graft: bone marrow mesenchymal stem cells-derived neural stem cells and patient's autoimmune T cells<br>-Procedure: arterial infusion	A Vojta and Bobath neurorehabilitation program
2005 Feron F.[148], Mackay-Sim A.[149]<br>Griffith University, Brisbane, Australia<br>Phase I / IIa, N = 6	-Graft: olfactory ensheathing cells<br>-Procedure: injected into the region of damaged spinal cord	No description about rehabilitation
2001 Wirth ED3rd.[147], 2001 Thompson FJ.[146]<br>University of Florida College of Medicine, Florida, US<br>Phase of clinical trial: not described, N = 2	-Graft: fetal spinal cord tissue<br>-Procedure: transplanted in syringomyelia	No description about rehabilitation<br>Transferred to rehabilitation department 18 days post transplantation

Abbreviations

AIS: ASIA impairment scale, BWSTT: body weight-supported treadmill training, FES: functional electrical stimulation, SCI: spinal cord injury

Supplementary references

104. Thornton, M.A. et al. Evidence of axon connectivity across a spinal cord transection in rats treated with epidural stimulation and motor training combined with olfactory ensheathing cell transplantation. *Exp Neurol* **309**, 119-133 (2018).
105. Dugan, E.A. & Shumsky, J.S. A combination therapy of neural and glial restricted precursor cells and chronic quipazine treatment paired with passive cycling promotes quipazine-induced stepping in adult spinalized rats. *J Spinal Cord Med* **38**, 792-804 (2015).
106. Carvalho, K.A. et al. Functional outcome of bone marrow stem cells (CD45(+)/CD34(-)) after cell therapy in acute spinal cord injury: in exercise training and in sedentary rats. *Transplant Proc* **40**, 847-9 (2008).
107. Lynskey, J.V. et al. Delayed intervention with transplants and neurotrophic factors supports recovery of forelimb function after cervical spinal cord injury in adult rats. *J Neurotrauma* **23**, 617-34 (2006).
108. Ruitenberg, M.J. et al. NT-3 expression from engineered olfactory ensheathing glia promotes spinal sparing and regeneration. *Brain* **128**, 839-53 (2005).
109. Keyvan-Fouladi, N., Raisman, G. & Li, Y. Functional repair of the corticospinal tract by delayed transplantation of olfactory ensheathing cells in adult rats. *J Neurosci* **23**, 9428-34 (2003).
110. Sharma, A. et al. Intrathecal transplantation of autologous bone marrow mononuclear cells in patients with sub-acute and chronic spinal cord injury: An open-label study. *Int J Health Sci (Qassim)* **14**, 24-32 (2020).
111. Anderson, K.D. et al. Safety of Autologous Human Schwann Cell Transplantation in Subacute Thoracic Spinal Cord Injury. *J Neurotrauma* **34**, 2950-2963 (2017).
112. Liu, J. et al. Clinical analysis of the treatment of spinal cord injury with umbilical cord mesenchymal stem cells. *Cytotherapy* **15**, 185-91 (2013).
113. Yoon, S.H. et al. Complete spinal cord injury treatment using autologous bone marrow cell transplantation and bone marrow stimulation with granulocyte macrophage-colony stimulating factor: Phase I/II clinical trial. *Stem Cells* **25**, 2066-73 (2007).
114. Rabinovich, S.S. et al. Transplantation treatment of spinal cord injury patients. *Biomed Pharmacother* **57**, 428-33 (2003).
115. Knoller, N. et al. Clinical experience using incubated autologous macrophages as a treatment for complete spinal cord injury: phase I study results. *J Neurosurg Spine* **3**, 173-81 (2005).
116. Shin, J.C. et al. Clinical Trial of Human Fetal Brain-Derived Neural Stem/Progenitor Cell Transplantation in Patients with Traumatic Cervical Spinal Cord Injury. *Neural Plast* **2015**, 630932 (2015).
117. Karamouzian, S., Nematollahi-Mahani, S.N., Nakhaee, N. & Eskandary, H. Clinical safety and primary efficacy of bone marrow mesenchymal cell transplantation in subacute spinal cord injured patients. *Clin Neurol Neurosurg* **114**, 935-9 (2012).
118. Chhabra, H.S. et al. Autologous bone marrow cell transplantation in acute spinal cord injury--an Indian pilot study. *Spinal Cord* **54**, 57-64 (2016).
119. Satti, H.S. et al. Autologous mesenchymal stromal cell transplantation for spinal cord injury: A Phase I pilot study. *Cytotherapy* **18**, 518-22 (2016).
120. Hur, J.W. et al. Intrathecal transplantation of autologous adipose-derived mesenchymal stem cells for treating spinal cord injury: A human trial. *J Spinal Cord Med* **39**, 655-664 (2016).
121. Bansal, H. et al. Autologous Bone Marrow-Derived Stem Cells in Spinal Cord Injury. *J Stem Cells* **11**, 51-61 (2016).
122. Xiao, Z. et al. Significant Improvement of Acute Complete Spinal Cord Injury Patients Diagnosed by a Combined Criteria Implanted with NeuroRegen Scaffolds and Mesenchymal Stem Cells. *Cell Transplant* **27**, 907-915 (2018).
123. Pal, R. et al. Ex vivo-expanded autologous bone marrow-derived mesenchymal stromal cells in human spinal cord injury/paraplegia: a pilot clinical study. *Cytotherapy* **11**, 897-911 (2009).
124. Attar, A. et al. An attempt to treat patients who have injured spinal cords with intralesional implantation of concentrated autologous bone marrow cells. *Cytotherapy* **13**, 54-60 (2011).
125. Kumar, A.A., Kumar, S.R., Narayanan, R., Arul, K. & Baskaran, M. Autologous bone marrow derived mononuclear cell therapy for spinal cord injury: A phase I/II clinical safety and primary efficacy data. *Exp Clin Transplant* **7**, 241-8 (2009).
126. Saito, F. et al. Spinal cord injury treatment with intrathecal autologous bone marrow stromal cell transplantation: the first clinical trial case report. *J Trauma* **64**, 53-9 (2008).
127. Saito, F. et al. Administration of cultured autologous bone marrow stromal cells into cerebrospinal fluid in spinal injury patients: a pilot study. *Restor Neurol Neurosci* **30**, 127-36 (2012).
128. Jones, L.A. et al. A phase 2 autologous cellular therapy trial in patients with acute, complete spinal cord injury: pragmatics, recruitment, and demographics. *Spinal Cord* **48**, 798-807 (2010).
129. Lammertse, D.P. et al. Autologous incubated macrophage therapy in acute, complete spinal cord injury: results of the phase 2 randomized controlled multicenter trial. *Spinal Cord* **50**, 661-71 (2012).
130. Gant, K.L. et al. Phase 1 Safety Trial of Autologous Human Schwann Cell Transplantation in Chronic Spinal Cord Injury. *J Neurotrauma* (2021).
131. Yao, L. et al. Human umbilical cord blood stem cell transplantation for the treatment of chronic spinal cord injury: Electrophysiological changes and long-term efficacy. *Neural Regen Res* **8**, 397-403 (2013).
132. Iwatsuki, K. et al. A Pilot Clinical Study of Olfactory Mucosa Autograft for Chronic Complete Spinal Cord Injury. *Neurol Med Chir (Tokyo)* **56**, 285-92 (2016).
133. Oraee-Yazdani, S. et al. Co-transplantation of autologous bone marrow mesenchymal stem cells and Schwann cells through cerebral spinal fluid for the treatment of patients with chronic spinal cord injury: safety and possible outcome. *Spinal Cord* **54**, 102-9 (2016).
134. El-Kheir, W.A. et al. Autologous bone marrow-derived cell therapy combined with physical therapy induces functional improvement in chronic spinal cord injury patients. *Cell Transplant* **23**, 729-45 (2014).
135. Kishk, N.A. et al. Case control series of intrathecal autologous bone marrow mesenchymal stem cell therapy for chronic spinal cord injury. *Neurorehabil Neural Repair* **24**, 702-8 (2010).

136. Mendonca, M.V. et al. Safety and neurological assessments after autologous transplantation of bone marrow mesenchymal stem cells in subjects with chronic spinal cord injury. *Stem Cell Res Ther* **5**, 126 (2014).
137. Dai, G. et al. Transplantation of autologous bone marrow mesenchymal stem cells in the treatment of complete and chronic cervical spinal cord injury. *Brain Res* **1533**, 73-9 (2013).
138. Derakhshanrad, N. et al. Safety of intramedullary autologous peripheral nerve grafts for post-rehabilitated complete motor spinal cord injuries: a phase I study. *Acta Med Iran* **51**, 842-54 (2013).
139. Saberi, H. et al. Safety of intramedullary Schwann cell transplantation for postrehabilitation spinal cord injuries: 2-year follow-up of 33 cases. *J Neurosurg Spine* **15**, 515-25 (2011).
140. Lima, C. et al. Olfactory mucosa autografts in human spinal cord injury: a pilot clinical study. *J Spinal Cord Med* **29**, 191-203; discussion 204-6 (2006).
141. Cheng, H. et al. Clinical observation of umbilical cord mesenchymal stem cell transplantation in treatment for sequelae of thoracolumbar spinal cord injury. *J Transl Med* **12**, 253 (2014).
142. Goni, V.G. et al. Safety profile, feasibility and early clinical outcome of cotransplantation of olfactory mucosa and bone marrow stem cells in chronic spinal cord injury patients. *Asian Spine J* **8**, 484-90 (2014).
143. Chernykh, E.R. et al. Application of autologous bone marrow stem cells in the therapy of spinal cord injury patients. *Bull Exp Biol Med* **143**, 543-7 (2007).
144. Wang, S. et al. Autologous Olfactory Lamina Propria Transplantation for Chronic Spinal Cord Injury: Three-Year Follow-Up Outcomes From a Prospective Double-Blinded Clinical Trial. *Cell Transplant* **25**, 141-57 (2016).
145. Chen, L. et al. A prospective randomized double-blind clinical trial using a combination of olfactory ensheathing cells and Schwann cells for the treatment of chronic complete spinal cord injuries. *Cell Transplant* **23 Suppl 1**, S35-44 (2014).
146. Thompson, F.J. et al. Neurophysiological assessment of the feasibility and safety of neural tissue transplantation in patients with syringomyelia. *J Neurotrauma* **18**, 931-45 (2001).
147. Wirth, E.D., 3rd et al. Feasibility and safety of neural tissue transplantation in patients with syringomyelia. *J Neurotrauma* **18**, 911-29 (2001).
148. Feron, F. et al. Autologous olfactory ensheathing cell transplantation in human spinal cord injury. *Brain* **128**, 2951-60 (2005).
149. Mackay-Sim, A. et al. Autologous olfactory ensheathing cell transplantation in human paraplegia: a 3-year clinical trial. *Brain* **131**, 2376-86 (2008).
150. Cristante, A.F. et al. Stem cells in the treatment of chronic spinal cord injury: evaluation of somatosensitive evoked potentials in 39 patients. *Spinal Cord* **47**, 733-8 (2009).
151. Ra, J.C. et al. Safety of intravenous infusion of human adipose tissue-derived mesenchymal stem cells in animals and humans. *Stem Cells Dev* **20**, 1297-308 (2011).
152. Wu, J. et al. Clinical observation of fetal olfactory ensheathing glia transplantation (OEGT) in patients with complete chronic spinal cord injury. *Cell Transplant* **21 Suppl 1**, S33-7 (2012).
153. Frolov, A.A. & Bryukhovetskiy, A.S. Effects of hematopoietic autologous stem cell transplantation to the chronically injured human spinal cord evaluated by motor and somatosensory evoked potentials methods. *Cell Transplant* **21 Suppl 1**, S49-55 (2012).
154. Rao, Y. et al. Clinical application of olfactory ensheathing cells in the treatment of spinal cord injury. *J Int Med Res* **41**, 473-81 (2013).
155. Al-Zoubi, A. et al. Transplantation of purified autologous leukapheresis-derived CD34+ and CD133+ stem cells for patients with chronic spinal cord injuries: long-term evaluation of safety and efficacy. *Cell Transplant* **23 Suppl 1**, S25-34 (2014).
156. Vaquero, J. et al. Repeated subarachnoid administrations of autologous mesenchymal stromal cells supported in autologous plasma improve quality of life in patients suffering incomplete spinal cord injury. *Cytotherapy* **19**, 349-359 (2017).
157. Vaquero, J. et al. An approach to personalized cell therapy in chronic complete paraplegia: The Puerta de Hierro phase I/II clinical trial. *Cytotherapy* **18**, 1025-1036 (2016).
158. Levi, A.D. et al. Emerging Safety of Intramedullary Transplantation of Human Neural Stem Cells in Chronic Cervical and Thoracic Spinal Cord Injury. *Neurosurgery* **82**, 562-575 (2018).
159. Zurita, M., Aguayo, C., Bonilla, C., Rodriguez, A. & Vaquero, J. Perilesional intrathecal administration of autologous bone marrow stromal cells achieves functional improvement in pigs with chronic paraplegia. *Cytotherapy* **15**, 1218-27 (2013).
160. Penha, E.M. et al. Clinical neurofunctional rehabilitation of a cat with spinal cord injury after hemilaminectomy and autologous stem cell transplantation. *Int J Stem Cells* **5**, 146-50 (2012).
